# Visual Scanning Training for Neglect after Stroke with and without a Computerized Lane Tracking Dual Task

**DOI:** 10.3389/fnhum.2013.00358

**Published:** 2013-07-10

**Authors:** M. E. van Kessel, A. C. H. Geurts, W. H. Brouwer, L. Fasotti

**Affiliations:** ^1^Donders Institute for Brain, Cognition and Behaviour, Radboud University Nijmegen, Nijmegen, Netherlands; ^2^Medisch Spectrum Twente Hospital Group, Enschede, Netherlands; ^3^Radboud University Nijmegen Medical Centre, Nijmegen, Netherlands; ^4^Department of Neurology, University Medical Center Groningen, Groningen, Netherlands; ^5^Department of Psychology, University of Groningen, Groningen, Netherlands; ^6^Medical Rehabilitation Centre Groot Klimmendaal/SIZA Support and Rehabilitation, Arnhem, Netherlands

**Keywords:** hemineglect, spatial attention, divided attention, virtual reality, driving simulator

## Abstract

Neglect patients typically fail to explore the contralesional half-space. During visual scanning training, these patients learn to consciously pay attention to contralesional target stimuli. It has been suggested that combining scanning training with methods addressing non-spatial attention might enhance training results. In the present study, a dual task training component was added to a visual scanning training (i.e., Training di Scanning Visuospaziale – TSVS; Pizzamiglio et al., 1990). Twenty-nine subacute right hemisphere stroke patients were semi-randomly assigned to an experimental (*N* = 14) or a control group (*N* = 15). Patients received 30 training sessions during 6 weeks. TSVS consisted of four standardized tasks (digit detection, reading/copying, copying drawings, and figure description). Moreover, a driving simulator task was integrated in the training procedure. Control patients practiced a single lane tracking task for 2 days a week during 6 weeks. The experimental group was administered the same training schedule, but in weeks 4–6 of the training, the TSVS digit detection task was combined with lane tracking on the same projection screen, so as to create a dual task (computerized visual reaction time task designed for training). Various neglect tests and driving simulator tasks were administered before and after training. No significant group and interaction effects were found that might reflect additional positive effects of dual task training. Significant improvements after training were observed in both groups taken together on most assessment tasks. Ameliorations were generally not correlated to post-onset time, but spontaneous recovery, test–retest variability, and learning effects could not be ruled out completely, since these were not controlled for. Future research might focus on increasing the amount of dual task training, the implementation of progressive difficulty levels in driving simulator tasks, and further exploration of relationships between dual task training and daily functioning.

## Introduction

Visuospatial neglect is defined as “a disorder whereby a patient fails to explore the half-space contralateral to the cerebral lesion” (Heilman et al., 1993). To explain the deficit underlying this disorder, various theories have been formulated, like attentional, representational, and cerebral balance theories (see Kerkhoff, 2001 for a review). Corbetta and Shulman (2011) suggest that neglect results from a dysfunction of the distributed and interacting cortical networks responsible for the control of both spatial and non-spatial attention processes. For instance, neglect symptoms have been shown to vary with arousal and sustained or vigilant attention (Robertson et al., 1997; Samuelsson et al., 1998; Robertson, 2001) as well as with task complexity (Deouell et al., 2005; Vuillemier et al., 2008).

Neglect occurs more often after right hemisphere (RH) than after left hemisphere (LH) stroke. Reported rates of occurrence vary widely as a result of a number of factors, including assessment method and time post stroke (see Bowen et al., 1999 for a review). Also, large within-patient variability in test performance is reported. Machner et al. (2012) administered the Bells test, a symbol cancelation and a line bisection task on five consecutive days to 15 neglect patients. They observed large day-to-day variability, indicating that five more or less omissions on the Bells test and deviations of plus or minus 16 mm in the line bisection task could be due to test or within-patient variability, rather than indicating a reliable change of neglect severity. Similar results have been reported by Bailey et al. (2004).

Spontaneous recovery of neglect is mostly reported in the first weeks after stroke (Ferro et al., 1999; Appelros et al., 2004b; Jehkonen et al., 2007). However, a recent study of Nijboer et al. (in press) reports significant spontaneous recovery up to 14 weeks after stroke. Farnè et al. (2004) also report changes in the performance of neglect tasks until at least 3 months after stroke. The presence of neglect is generally associated with poor functional outcome after stroke (Jehkonen et al., 2006; DiMonaco et al., 2011; Vossel et al., 2012). Nijboer et al. (in press) point out that 40% of the neglect patients still show visuospatial neglect 1 year after stroke, indicating that rehabilitation of this disorder is of great importance.

Several interventions aimed at reducing neglect symptoms have been described, like visual scanning training, prism adaptation, limb activation training, and non-invasive brain stimulation techniques (see Zoccolotti et al., 2011; Kerkhoff and Schenk, 2012; Fasotti and Van Kessel, in press, for reviews). In a Cochrane review excluding all studies that were not considered properly randomized controlled trials, Bowen and Lincoln (2007) conclude that there is insufficient evidence to support the effectiveness of specific cognitive rehabilitation approaches for reducing disabilities due to neglect (see also Rohling et al., 2009; Paci et al., 2010). However, in two reviews of cognitive rehabilitation, Cicerone et al. (2000, 2005) recommend visual scanning training as a practice standard for the treatment of neglect. Also, in a meta-analysis of the reviews by Cicerone et al., Rohling et al. (2009) report a medium to large effect of visuospatial training. In an extensive review of 18 different treatments for neglect and their rationales, in which not only randomized controlled trials but also multiple baseline single case studies were included, Luauté et al. (2006) conclude that for 6 of the available methods there is some evidence for clinical relevant training effects, visual scanning training being the most extensively evaluated training method.

Visual scanning training was originally introduced by Diller and Weinberg (1977) and further developed and described by Pizzamiglio et al. (1990, 1992) (see Pizzamiglio et al., 2006 for a review). This type of training stems from the observation that neglect patients generally show very limited attention and exploration behavior toward the contralesional hemispace. The aim of training is to improve visual scanning behavior, i.e., to encourage neglect patients to actively and consciously pay attention to stimuli on the contralesional side. In the original training protocol by Pizzamiglio et al. (1990) (Training di Scanning Visuospaziale – TSVS), four standardized training tasks are used, i.e., a computerized digit detection task projected on a large screen, figure copying, picture exploring, and reading and writing tasks. Contralesional exploration behavior is encouraged by means of operant conditioning techniques (i.e., reinforcement of correct scanning movements) and repeated training of the use of compensatory strategies (for instance using a contralesional anchor and systematically starting to scan from this point and controlling one’s performance starting from the contralesional side before finishing an activity). Guidelines for the use and gradual reduction of various stimulation methods and cues are provided. Moreover, in order to increase their awareness of the deficit, patients are given concrete feedback about their performance.

Significantly increased scores on paper-and-pencil tasks as well as on a semi-structured observation scale (Zoccolotti et al., 1992) were found after TSVS (Pizzamiglio et al., 1992; Antonucci et al., 1995). The authors stress that the duration and intensity of the training (40 h during 8 weeks) plays an important role in the attainment of positive results. Moreover, the gradual and systematic increase in difficulty levels of the materials and the reduction of feedback seem important ingredients of the training leading to improvement. Positive training results were replicated by Paolucci et al. (1996), who found improvements in test performance as well as in functional status linked to the timing of the training and additional to general rehabilitation. Despite these generally positive results, a large variability in patients’ benefits from TSVS has also been observed in each of the abovementioned studies. It is unclear why some patients benefit from the training while others do not. One factor seems to be the improvement of the patients’ awareness of deficit (Pizzamiglio et al., 1992). However, often it is not possible to predict whether improved awareness may be expected in an individual patient as a result of the training. In addition, since neglect may occur after lesions in different regions of the brain (see for instance Karnath et al., 2004), lesion site might also play a role in the variability of training effects.

As various authors (Pizzamiglio et al., 2006; Saevarsson et al., 2011) point out, individual variability in training results has led to the question whether training effectiveness can be improved by combining interventions. Until now, positive training results were found in both conditions in a study comparing regular TSVS with TSVS plus additional optokinetic stimulation (Pizzamiglio et al., 2004). However, no differences were observed between conditions. Luauté et al. (2006) also recommend the evaluation of combinations of existing methods. More specifically, these authors suggest that effective treatments be combined with techniques aiming at processes that contribute to the clinical manifestation of neglect (for example non-spatial attention and working memory) to further enhance training effects. Moreover, in another TSVS evaluation study, Piccardi et al. (2006) investigated whether TSVS might result in improved performances on various neglect and non-neglect measures (i.e., measures of vigilance, alertness, and attentional control/response inhibition). TSVS training effects were observed on neglect measures but not on non-spatial attention tasks. Therefore, Pizzamiglio et al. (2006) point out that in the rehabilitation of neglect, care must be taken to also treat non-spatial disorders.

In the present study, an attempt is made to further extend the scope of standardized TSVS by combining it with additional dual task training. The use of dual tasks in neglect may be pre-eminently useful because of the association between spatial and non-spatial attentional processes in this disorder. Robertson and Frasca (1992), for instance, assume that neglect patients are particularly vulnerable to a deterioration of performance in the face of additional attentional load because of this association. Robertson and Manly (2004) suggested that it is possible to detect the presence of well-compensated or even “recovered” neglect by increasing attentional load. This can be accomplished by means of a dual task. In line with this idea, it was found that computerized dual tasks elicit more contralesional omissions (Bonato et al., 2012, 2013) and slower contralesional reaction times (RTs) (Deouell et al., 2005) than single paper-and-pencil tasks. Moreover, clearly asymmetric task performance in the computerized dual tasks even occurred in some patients showing no signs of neglect in paper-and-pencil tasks. Thus, computer-based dual tasks, even though not always showing resemblance to contexts of daily living, have high diagnostic potential in the assessment of neglect and its recovery (Schendel and Robertson, 2002; Bonato and Deouell, 2013).

Furthermore, various authors describe that deficits in non-spatial attentional processes not only occur in association with neglect (for instance in the case of impaired arousal). Non-spatial attention processes involved in the exertion of top-down influence on lower level spatial perception may also play an important underlying role in this disorder (Corbetta et al., 2005; Vuillemier et al., 2008). Bartolomeo and Chokron (2002), for instance, suggest that a basic mechanism leading to neglect behavior is an impaired exogenous orienting toward left-sided targets. Nevertheless, patients may be able to compensate for their deficit by means of endogenous attentional processes, that may be spared but slowed in neglect. The ability to successfully compensate for neglect symptoms might thus depend on the patients’ capacities to gain attentional control over their scanning behavior.

Neglect is often associated with frontoparietal damage in the RH (Farnè et al., 2004; Committeri et al., 2007) or in the white matter connecting parietal and frontal areas (Bartolomeo et al., 2007, 2012). According to Corbetta and Shulman (2011), lesions in the RH that cause neglect impair non-spatial functions mediated by a ventral frontoparietal attention network. This impairment may in turn induce abnormalities in an anatomically linked dorsal frontoparietal network that controls spatial attention. Singh-Curry and Husain (2009) point out that a frontoparietal system might allow the flexible reconfiguration of behavior between maintaining attentive control and responding to salient stimuli. Dual tasks might then not only generally increase attentional load, but might address this frontoparietal system more specifically.

Thus, dual tasks might not only appeal to attentional capacity, but also to the control over attention. Patients’ performances on these tasks could be indicative for their abilities to compensate for neglect (Van Kessel et al., 2013). This raises the question whether these tasks might also be used as a training tool. As Robertson and Manly (2004) point out, the demands on neglect patients’ impaired abilities in maintaining corrective “top-down” control over spatial attention might be minimized by attempting to train these corrective strategies to a point where they become more habitual. TSVS training is aimed at the conscious compensation for spatial attention deficits and thus appeals to top-down attentional control. Combining TSVS with additional dual task training might provide tools for accomplishing a higher degree of automation of scanning strategies and contribute to the enhancement of training results.

To investigate the additional value of dual task training, in the present study, a computerized visual RT task designed for training (CVRT-TR) will be used. The CVRT-TR was designed on the basis of two diagnostic tasks, i.e., a single and a dual CVRT task (CVRT and CVRT-D, respectively). These assessment tasks had been previously used to investigate spatial and non-spatial attention processes in neglect (Van Kessel et al., 2010, 2013). In concordance with the abovementioned findings of Deouell et al. (2005) and Bonato et al. (2012, 2013), more patients were classified as neglect patients by using RT asymmetries on the CVRT than by using scores on the Behavioral Inattention Test (BIT; Wilson et al., 1987). Moreover, the results suggested that some patients with defective RT asymmetries but normal BIT scores might compensate for their lateralized deficit in paper-and-pencil tasks. These patients might have engaged intact non-spatial attentional processes, especially attentional control (Van Kessel et al., 2010). When single (CVRT) and dual (CVRT-D) task performance were compared (Van Kessel et al., 2012), a clear increase in RT asymmetries between CVRT and CVRT-D was observed. Half of the patients meeting the BIT criterion for neglect showed increased RT asymmetries from CVRT to CVRT-D. Moreover, two LH and RH patients without neglect symptoms on the BIT and CVRT showed significantly increased asymmetries in the CVRT-D. This fostered the idea of an emergence of subtle neglect under increased attentional load.

In the CVRT-TR, a large screen driving simulation task was added to the computerized digit detection task used in the standardized TSVS protocol (Pizzamiglio et al., 1990). Thus, a dual task was created that can be used for training patients. The CVRT-TR could be referred to as a virtual reality (VR) task. Other VR methods include desktop simulator tasks or head-mounted devices. Recently, different kinds of VR tasks have been applied in the assessment and observation of neglect patients (Broeren et al., 2007; Buxbaum et al., 2008, 2012; Jannink et al., 2009; Kim et al., 2010; Fordell et al., 2011). Buxbaum et al. (2008, 2012) describe a virtual reality lateralized attention task (VRLAT) in which patients had to navigate through a VR environment while seated in front of a flat screen display in a powered wheelchair treadmill. These patients were asked to name objects projected on both sides of the road. Neglect symptoms were detected in more patients by using the VRLAT, compared to paper-and-pencil tasks. Moreover, left-sided collisions on the VRLAT showed significant correlations with real-world left-sided collisions.

Virtual reality tasks are also used as a rehabilitation tool (see Tsirlin et al., 2009 for a recent review). VR training in neglect is mostly aimed at improving performance on the task that is simulated, for instance navigating through a real-life wheelchair obstacle course (Webster et al., 2001) or street crossing (Katz et al., 2005). In an alertness training program used by Thimm et al. (2006), patients had to “drive” a simulated car or motorcycle as quickly as possible and avoid crashing into obstacles that appeared suddenly on the screen. After 3 weeks of training, both alertness and neglect deficits were significantly reduced. However, 4 weeks after the end of training, neglect symptoms had returned to the pre-training level. Finally, Akinwuntan et al. (2010) observed no differences between stroke patients receiving either simulator-based driving-related training or non-computer-based cognitive training over 5 weeks. In their RCT, both groups showed similar improvement after training on a test of driving-related visual attention skills.

Not only are VR techniques suitable to simulate daily activities, but in doing so, tasks can be created that allow for the combined training of visuospatial and non-spatial attention. In the present study, it will be investigated whether the effectiveness of the standardized TSVS protocol (Pizzamiglio et al., 1990, 1992) might be further enhanced using the CVRT-TR. In the CVRT-TR, patients are enabled to additionally practice their acquired scanning strategies while performing a secondary task. It is hypothesized that training patients with this task could contribute to an enhancement in TSVS training results and better performance on various diagnostic tasks for neglect.

## Materials and Methods

### Participants

Patients with a first intracerebral infarction or hemorrhage admitted for clinical multidisciplinary rehabilitation to one of four local rehabilitation centers in the Netherlands were eligible for this study. Over a period of 2 years, 53 RH patients showing neglect symptoms as observed by their therapists and/or found in early neuropsychological screening, were referred for further assessment. This assessment was aimed at investigating whether TSVS and inclusion in the present study would be indicated. Tests were performed at least 8 weeks post-onset to minimize the role of spontaneous recovery. Six patients in the control group and 8 patients in the experimental group could be considered chronic neglect patients, since they had post-onset times of more than 3 months. Patients with omission scores above cut-off on at least three of the paper-and-pencil neglect tests and one of the observational scales (all listed below) were asked to participate in the present study. Patients with visual field deficits as observed by means of Donders’ confrontation method were excluded. A total of 29 patients were included. All subjects gave informed consent to participate in this study and research was completed in accordance with the Declaration of Helsinki. In Table 1, medical and demographic data of the subjects are presented.

**Table 1:** **Medical and demographic data for both patient groups**.

	Control (*N* = 15)	Experimental (*N* = 14)
Sex (male/female)	10/5	7/7
Mean age (SD)	59.07 (6.08)	61.86 (7.75)
Range	48–71	52–77
Days post onset (SD)	157.60 (117.16)	140.57 (133.56)
Range	63–431	57–569

Patients were assigned to the experimental or control group using block semi-randomization. Of every four consecutive patients, the first two (in case these two were assigned to the same group) or three (if the first two patients each were assigned to a different group) were randomly allocated to one of the groups. The other(s) were classified in such a way that within every block of four, two patients were in the experimental and two in the control group.

### Pre- and post-training assessments

Patients were administered various neglect tasks (see below) on two separate days within 1 week. The first session included the paper-and-pencil and driving simulator tasks and lasted for approximately 1 h. The semi-structured scales were administered on a second day, because another room (kitchen of the occupational therapy department) was necessary to administer these tasks. When a patient was included, training started 1 or 2 weeks after the first assessment. Post training assessments were scheduled 1 or 2 weeks after the end of the training.

#### Paper-and-pencil neglect tests

##### Line cancelation

Patients were asked to cross out 21 lines (2.5 cm) printed on a A3 sheet of paper (Albert, 1973). The occurrence of one or more omissions was considered as indicative for neglect.

##### Letter cancelation

Patients were instructed to cross out 104 uppercase “H”s interspersed among 208 distractor characters (Diller and Weinberg, 1977). All characters were printed in six horizontal lines on a A3 sheet of paper. Five or more omissions and a difference of two or more between contralesional and ipsilesional omissions were considered as indicative for neglect.

##### Bells test

Thirty-five bell-shaped figures, interspersed among 280 distractor figures and printed on a A4 sheet of paper, had to be crossed out (Gauthier et al., 1989). Four or more omissions were considered as indicative for neglect.

##### Line bisection

Patients were asked to bisect 20 horizontal lines (printed on a A4 sheet of paper) by placing a pencil mark as close to the center of the line as possible (Schenkenberg et al., 1980). Two or more omitted lines were considered as indicative for neglect.

##### Word reading task

Patients were asked to read aloud 165 words and non-words, each printed on a different sheet of A4 paper (after Làdavas et al., 1997). All words consisted of three syllables and were composed of 6–11 letters. Fifty-five words were used in their natural form. Moreover, in every word two letters were replaced within the first syllable in one condition (left non-word) and within the last in a third condition (right non-word). All words (55) and non-words (110) were presented in random order. RH neglect patients tend to misread the first syllables. An index score was computed in which the difference between left and right errors was divided by the sum of left and right errors. Ignoring some letters or the complete first syllable, or (in case of left non-words) reading the original word as if no letters had been replaced in the first syllable were considered errors.

##### Grey scales

Twenty-six sheets of paper (A4, landscape) were presented to the patients (Tant et al., 2002). A pair of vertically aligned horizontal rectangular bars of equal length was printed on each page. The bars were filled with continuous scales of different gray shades varying from black to white at the extremes. The upper and lower bar of each pair were mirrored copies of each other. Hence, one of the gray scales was black on the left and white on the right and the other exactly the opposite. Pairs of stimuli of different lengths were randomly used. Patients were asked to judge which (top or bottom) bar of each pair appeared darker overall. RH neglect patients tend to show extreme rightward biases (consistently choosing bars that are black on the extreme right). An index score was computed, in which the difference between rightward and leftward biased responses was divided by 26.

##### Baking tray task

In this task, patients were asked to equally distribute 16 blocks (4 cm × 4 cm) on a “baking tray,” i.e., a 75 cm × 100 cm board (Tham and Tegnér, 1996; Appelros et al., 2004a). A difference of two or more between the numbers of blocks placed left and right was considered as indicative for neglect. An index score was computed, in which the difference between the numbers of blocks placed right and left was divided by 16.

#### Observation scales and subjective questionnaire

##### Semi-structured scale for the evaluation of extrapersonal neglect

This task consisted of four subscales (serving tea, dealing cards, description of the environment, and of three large pictures), performed in the presence of the examiner and two additional persons seated at the left and right side of the table (Zoccolotti et al., 1992). Six scores for the extent of asymmetric performance were given on 0–3 point scales, so that the maximum total score of 18 indicated severe asymmetries on all subscales. A total score of 3 or more was considered as indicative for neglect.

##### Semi-structured scale for the evaluation of personal neglect

The patient was asked to show how he/she would comb his/her hair, using a razor (male) or powder her face (female) and putting on glasses (Zoccolotti et al., 1992). Three asymmetry scores were given on 0–3 point scales. A total score of 2 or more was considered as indicative for neglect.

##### Subjective neglect questionnaire

This questionnaire consisted of 19 items describing common problems associated with neglect (for instance bumping into door frames) (Towle and Lincoln, 1991). Patients were asked to indicate how frequently (1–5) each problem had occurred the last month. Thus, the minimum score of 19 indicated no reported problems, the maximum score was 95.

#### Driving simulator tasks

Three types of driving simulator tasks were used during the assessment. These were a single lane tracking task, a single target detection task, and a dual task consisting of both lane tracking and target detection (see also below). In all driving simulator tasks, patients were seated in front of a 2.13 m × 3.18 m projection screen at a distance of approximately 90 cm, thus creating a visual angle of approximately 110°. On the screen, a driving scene was projected. A steering wheel (Trust formula 1 race master) was fixed on a table in front of the participant and a white wooden board was placed on the table between the steering wheel and the projection screen, so as to prevent subjects from using the edges of the table as a spatial reference while driving.

##### Lane tracking

In the lane tracking task, a driving scene was projected on the same screen that was also used as a part of the standard TSVS training (e.g., large screen digit detection, see below). The simulated speed of the imaginary car was set at a constant 50 km/h. Patients were instructed to use the steering wheel to maintain the starting position in the middle of the right lane of the projected road, thereby compensating for what was indicated as “sidewind.” This was a continuous signal fluctuating from left to right in a fixed pattern created by superimposing three low frequency sinus movements. Thus, patients were continuously “blown” off track, either right- or leftward. Patients’ lateral positions during lane tracking were recorded every 15 s. Mean lateral position scores were computed from these values for each patient and the SD of the lateral position scores reflected the degree of oscillation.

##### Single detection task (CVRT)

In the CVRT, patients were asked to detect large rectangular dot patterns on one of three horizontal positions within a driving scene that was projected on the screen. RTs for left, middle, and right stimuli were recorded and asymmetries (i.e., difference scores) between left and right RTs were computed. Steering was not required.

##### Dual task (CVRT-D)

In the CVRT-D, lane tracking and CVRT dot pattern detection were combined to create a dual task. Lateral position and oscillation scores were computed together with RTs and RT asymmetries.

### Training tasks

A translated version of the original TSVS manual (Pizzamiglio et al., 1990) was used. This was slightly adapted for use in the present study. Most importantly, patients received 30 training sessions (5 days a week, a 1-h session each day, during 6 weeks) instead of the original 40 h. Moreover, some changes had been made in the order of the digit detection sequences. Guidelines as to the use and fading of cues were provided in the manual. By individually adjusting difficulty levels of the sequences and the use of cues, patients were offered systematic training. Training sessions consisted of four standard tasks and additional control or experimental tasks.

#### Standard training

##### Large screen digit detection

**Figure 1:**
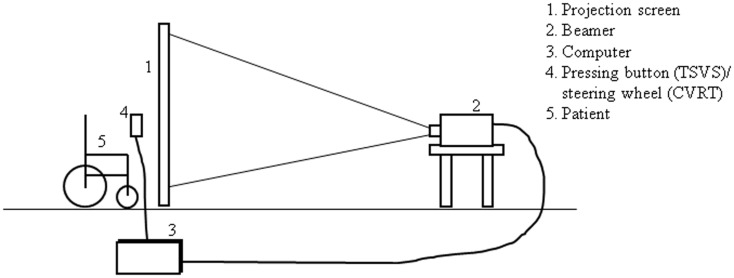
**TSVS visual scanning training set-up**.

Using a desktop computer and a projector, sequences of random digits (1–9) were projected from behind on a 3.18 m × 2.13 m screen. Each digit was projected at one of 48 (12 horizontal × 4 vertical) possible positions. Patients were seated in front of the screen, which was placed at approximately 90 cm from their eyes, so as to create a visual angle of the projection of around 110° horizontally and 70° vertically (see Figure 1 for training set-up). Patients were free to move their head and eyes. They were asked to name each digit and at the same time press a button as quickly as possible. Sequences of progressive difficulty levels were used, the easiest sequences progressing stepwise from right to left at the same height and the most difficult ones randomly alternating between all possible positions. Verbal cues (encouragement of the trainer to look further to the left) and non-verbal auditory cues (signal tones accompanying each digit) could be given.

In general, during the first weeks of training, patients were trained to perform leftward scanning movements. To this end, training sequences were used that facilitated directing and preserving attention (supported by active head movements) to the left side of the screen, i.e., progressing stepwise to and (later) from the left side.

In the second half of the training, patients were taught to “center” their scanning behavior, i.e., using their straight ahead as a departing point from which to make scanning movements to either the left or right side. This technique is aimed at achieving symmetry in left and right detection times. The use of verbal and auditory cues was gradually faded during the training.

##### Copying line drawings on a dot matrix

Patients were instructed to copy lines, connecting some points of a dot matrix placed on the left halve of a page, into an empty matrix on the right. Matrices varied from 4 to 20 points. The use of verbal and visual cues was progressively reduced.

##### Reading and copying training

Patients were asked to read and/or copy sentences and newspaper headlines of progressive difficulty levels (based on size and length as well as the number and spatial distribution of lines). The use of verbal and visual cues was progressively reduced.

##### Figure description

Patients were encouraged to describe all elements on pictures printed on A3-sized pages. Picture complexity gradually increased over a total of 45–60 pictures. The most simple pictures represented small numbers of centrally placed large objects that had to be counted. In the most complex pictures, figural elements or portions of text that were essential to capture the meaning of the depicted scene were placed at the extreme left side of the paper.

#### Additional tasks for the control and experimental conditions

In Table 2, the training schedules for the experimental and control groups are displayed, including the number of minutes per task for each session. As can be seen, from the second half of the training on, the two groups had different training schedules for 2 days a week. On Thursdays and Fridays, the TSVS large screen scanning task was (partly or as a whole) replaced by either the lane tracking or CVRT-TR task.

**Table 2:** **Training schedule for both groups**.

	Monday–Wednesday	Thursday–Friday	
Week 1–3	**Both conditions: TSVS**	**Both conditions: TSVS + lane tracking**
	Digit detection (30)	Digit detection (20)
	Copying drawings (15)	*Lane tracking task (15)*
	Reading/copying (10)	Copying drawings (15)
	Figure description (5)	Reading/copying (10)
Week 4–6		**Control condition: TSVS + lane tracking**	**Experimental condition: TSVS + dual task**
		Digit detection (20)	*CVRT-TR (35)*
		*Lane tracking task (15)*	Copying drawings (15)
		Copying drawings (15)	Reading/copying (10)
		Reading/copying (10)	

In this training schedule, on Mondays to Wednesdays, the standardized TSVS protocol (Pizzamiglio et al., 1990) was practiced. The division of tasks on Thursdays and Fridays was based on clinical experience. It was chosen for two reasons: first, driving simulator tasks were only added for 2 days a week since it was considered important that patients in both the control and experimental condition were allowed sufficient time to practice TSVS digit detection. Second, the CVRT-TR dual was only introduced from week 4 of the training because it was presumed that patients should first learn the centering technique as a requisite skill for an adequate execution of the dual task.

##### Lane tracking task

This task was also used as a part of the pre- and post-training assessment, see for details under Section “Driving Simulator Tasks.”

##### CVRT-TR dual task

**Figure 2:**
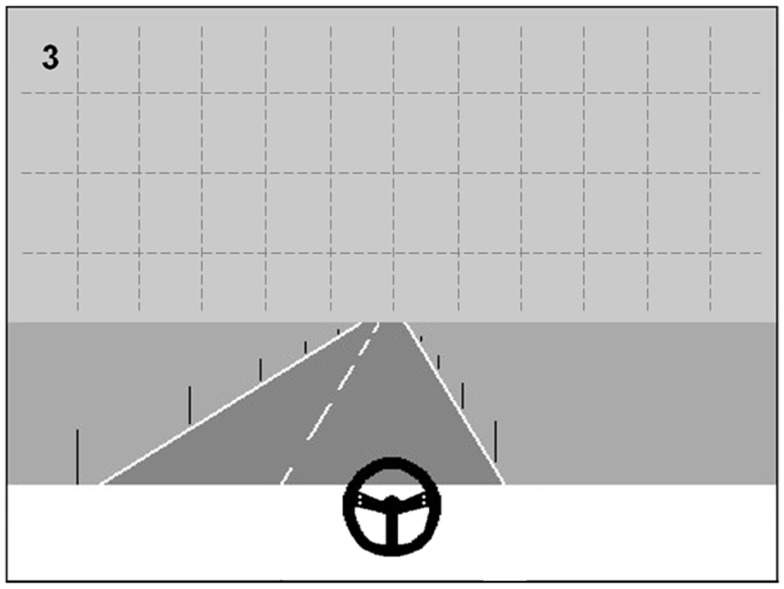
**CVRT-TR driving scene with digit projected at one of 48 possible locations**.

The CVRT-TR dual task was designed as a training counterpart of the CVRT-D, that was used as a diagnostic task in the present study (see Driving Simulator Tasks). Instead of the large rectangular dot patterns on three possible positions used in the CVRT-D, sequences from the TSVS large screen digit detection task were projected in the driving scene in CVRT-TR conditions. Thus, besides maintaining their driving position, patients were instructed to detect and name digits that were projected on the upper half of the screen at one of 48 possible locations (see Figure 2 for an example). The digit sequences that were projected were the same sequences that were used in the TSVS for training patients to “center” their scanning behavior (see also Standard Training) during the second half of the training. Thus, patients were enabled to further practice this centering technique during the CVRT-TR, by choosing to focus on the straight ahead (i.e., the road in front of them) and regularly performing scanning movements to the left or right to detect digits while driving.

### Data analysis

Severity of neglect before and after training was analyzed using non-parametric (Mann–Whitney *U*) tests. Mixed models analyses were performed for relevant measures with time (before vs. after training) as a within subjects factor and condition (control vs. experimental) as a between-subjects factor (*N* = 15, 14). Mixed Models is a procedure in which alternative estimators are used for the parameters of a variance-analytic design; it is claimed to be more robust to violations of assumptions which are crucial for the conventional ANOVA estimators. The procedure used here was restricted maximum likelihood estimators (REML). For the covariance structure, we opted for “unstructured” (see also Rietveld, 2005). The same procedure and covariance structure are used in all Mixed Models analyses reported throughout the Results section.

## Results

### Paper-and-pencil tasks

Patients’ performances on the administered paper-and-pencil tasks before and after training are shown in Table 3.

**Table 3:** **Mean scores and SDs on paper-and-pencil and driving measures before and after training for the control (C) and experimental (E) group**.

	Before training		After training		Before vs. after*
	C (*N* = 15)	E (*N* = 14)	C (*N* = 15)	E (*N* = 14)	Both groups (*N* = 29)
Line cancelation omissions (SD) cut-off: ≥1	1.53 (3.27)	2.07 (2.79)	0.40 (0.91)	0.71 (1.54)	*p* < 0.01
Letter cancelation omissions (SD) cut-off: ≥5, L vs. R ≥ 2	30.07 (29.23)	24.07 (24.15)	15.33 (20.11)	12.93 (21.55)	*p* < 0.001
Bells test omissions (SD) cut-off: ≥4	10.20 (6.84)	12.21 (8.83)	6.80 (5.13)	6.71 (7.52)	*p* < 0.005
Line bisection omissions (SD) cut-off: ≥2	1.53 (2.47)	2.43 (3.52)	0.67 (1.18)	2.21 (3.42)	ns
Reading errors (SD)	22.87 (27.28)	17.36 (22.38)	5.71 (4.82)	13.43 (11.59)	*p* < 0.005
Gray scales index (SD)	0.97 (0.10)	0.99 (0.03)	0.84 (0.32)	0.93 (0.17)	*p* < 0.05
Baking tray index (SD)	0.36 (0.59)	0.39 (0.55)	0.19 (0.57)	0.43 (0.40)	ns
Semi-structured scale extrapersonal (SD) cut-off: ≥3	6.33 (3.44)	6.79 (2.52)	3.07 (2.66)	2.71 (2.05)	*p* < 0.001
Semi-structured scale personal (SD) cut-off: ≥2	2.27 (1.58)	2.21 (2.61)	0.93 (1.10)	1.00 (0.96)	*p* < 0.005
Subjective neglect questionnaire (SD)	43.33 (13.54)	40.50 (11.11)	37.87 (11.90)	31.69 (9.46)	*p* < 0.005

*Significance level α = 0.05, Wilcoxon signed-rank tests for two related samples.

As can be seen in Table 3, patients in both groups taken together showed significantly improved performances on almost all paper-and-pencil tasks. However, Mann–Whitney *U* tests did not show significant differences between groups on either of these scores, neither before nor after training.

A Mixed Models analysis was performed for a combined total score computed from the numbers of omissions on the line and letter cancelation tasks and the Bells test. The value of the −2 Restricted Log Likelihood information criterion was 510.08. The number of omissions in the cancelation tasks had decreased significantly after training in both groups as a whole [*F*(1, 27) = 19.02, *p* < 0.001], but no significant group effect [*F*(1, 27) = 0.07) or time × group interaction [*F*(1, 27) = 0.02] was found.

A Mixed Models analysis was also performed for a total score computed from the semi-structured scales for extrapersonal and personal neglect. The value of the −2 Restricted Log Likelihood information criterion for this analysis was 283.21. In general, patients in both groups showed significantly milder neglect symptoms on the semi-structured scales after training [*F*(1, 27) = 68.13, *p* < 0.001], but again, no significant group effect [*F*(1, 27) = 0.002] or time × group interaction [*F*(1, 27) = 0.33] was found.

### Driving simulator data

Patients’ performances on the lane tracking, CVRT, and CVRT-D tasks before and after training are represented in Table 4.

**Table 4:** **Mean scores and SDs on driving measures before and after training for each group**.

			Before training		After training		Before vs. after*
			C (*N* = 15)	E (*N* = 14)	C (*N* = 15)	E (*N* = 14)	Both groups (*N* = 29)
Lateral position		Lane tracking (SD)	−214.00 (213.10)	−153.66 (153.77)	−131.15 (145.39)	−128.71 (120.26)	*p* < 0.05
		CVRT-D (SD)	−224.44 (209.29)	−181.36 (181.50)	−156.95 (170.56)	−111.03 (110.34)	*p* < 0.05
Oscillation		Lane tracking (SD)	−71.12 (39.51)	−89.08 (62.06)	−68.38 (41.44)	−80.00 (59.70)	ns
		CVRT-D (SD)	−64.49 (37.24)	−80.60 (49.15)	−63.26 (28.29)	−71.85 (38.11)	ns
Omissions		CVRT (SD)	−5.60 (5.37)	−2.69 (3.47)	−2.33 (3.70)	−1.83 (4.30)	*p* = 0.057
		CVRT-D (SD)	−6.40 (5.51)	−6.23 (6.39)	−5.27 (5.35)	−3.25 (5.45)	ns
RT	CVRT	Left (SD)	−1524.57 (1121.61)	−1737.53 (1047.30)	−1664.26 (1196.69)	−1349.18 (928.02)	*p* < 0.05
		Middle (SD)	−882.10 (677.78)	−864.59 (609.41)	−601.02 (275.94)	−853.43 (574.88)	ns
		Right (SD)	−733.04 (660.74)	−845.01 (471.01)	−616.55 (276.74)	−857.84 (556.25)	ns
RT	CVRT-D	Left (SD)	−2176.96 (1280.29)	−2105.93 (1460.54)	−1786.43 (1071.84)	−1759.81 (1154.38)	ns
		Middle (SD)	−884.51 (634.93)	−1106.57 (787.62)	−679.57 (297.99)	−987.30 (916.31)	*p* < 0.05
		Right (SD)	−860.14 (475.40)	−951.92 (649.09)	−660.23 (276.52)	−911.16 (542.13)	*p* = 0.058

*Significance level α = 0.05, Wilcoxon signed-rank tests for two related samples.

The results in the last column of Table 4 show that patients in both groups together had significantly improved on lateral positions in single as well as dual lane tracking after training. They also made less omissions and showed faster contralesional RTs in the CVRT as well as faster middle and ipsilesional RTs in the CVRT-D. However, again, Mann–Whitney *U* tests did not show significant differences between groups on any score, neither before nor after training.

A Mixed Models analysis was performed for left versus right RT asymmetries in the CVRT and CVRT-D. The values of the −2 Restricted Log Likelihood information criterion for these analysis were 753.34 and 710.29, respectively. No significant differences were found between asymmetries before and after training [CVRT: *F*(1, 24.7) = 0.09, CVRT-D: *F*(1, 18.1) = 1.32] or between groups [CVRT: *F*(1, 25.6) = 0.73, CVRT-D: *F*(1, 21.4) = 0.01]. Also, interaction effects were not significant [CVRT: *F*(1, 24.7) = 2.68, CVRT-D: *F*(1, 18.1) = 0.91]. It should be noted that since some patients omitted all left stimuli in the CVRT, CVRT-D, or both, this resulted in missing data for the RTs on this position. Therefore, varying degrees of freedom are reported. Moreover, as a result of the fact that only valid RTs were recorded, valid RTs might show an increase instead of a decrease in patients who after training did respond to stimuli they had omitted before.

### Correlations with post-onset times

To account for the possible role of spontaneous recovery, two-tailed Pearson correlations were computed between days post-onset on the one hand and pre- vs. post-training differences on the other. These correlations were calculated for all measures showing significant differences in pre- vs. post-training performances (see Tables 3 and 4). Similar correlations were also computed between the post-onset period (in days) and pre-training as well as post-training performances. Bonferroni Holm corrections for multiple correlations (12 correlations for pre-training, post-training and pre- vs. post-training differences) were performed. No significant correlations of any measure with post-onset period were found.

## Discussion

In the present study, a computerized dual task was added to a standardized TSVS training (Pizzamiglio et al., 1990, 1992) for neglect patients. In this manner, patients were trained to use visual scanning strategies in an attention demanding task. It was hypothesized that this might enhance the automation of scanning strategies and thus contribute to an improvement of training results. Twenty-nine RH neglect patients, quasi-randomly assigned to one of two additional driving simulator training conditions, received TSVS training for 5 days a week during 6 weeks. In both conditions, for 2 days a week, the TSVS large screen digit detection task was replaced by a driving simulator task. In the control condition, patients trained with a lane tracking task two times a week during 6 weeks. In the experimental condition, lane tracking was replaced by CVRT-TR dual task training in weeks 4–6 of the training.

The primary research question of the present study was whether dual task training could contribute to an improvement of TSVS training results, as measured by various neglect tasks. No significant group or interaction effects reflecting additional positive training effects were found in the experimental group compared with the control group. Several explanations for the absence of group or interaction effects reflect the shortcomings of the present study and give clues for future research.

First, the amount of training time has to be considered. In the present study, the difference between control and experimental training time was two periods of 35 min per week during 3 weeks. This amount of time may be too small to find differences between conditions. The present results suggest that all patients had trained enough to show some improvement on most of the paper-and-pencil tasks as well as the simplest driving simulator subtasks, i.e., lane tracking and the detection of left stimuli in the CVRT single task. However, no improvement or practice effects were observed on the more complex CVRT-D.

Given the absence of a no-treatment control group, it can not be excluded that improved performance on the assessment tasks is due to spontaneous recovery, test–retest learning effects, or an interaction between these factors. Nijboer et al. (in press), for instance, found spontaneous recovery occurring up to 14 weeks after onset, on several paper-and-pencil tasks. Computerized dual tasks like the CVRT-D used in our study may show a higher sensitivity, even to slight signs of spontaneous recovery. Therefore, in future research, we recommend the use of longer post-onset times as an inclusion criterion and/or the inclusion of a no-treatment control group. Nevertheless, no significant correlations were found between pre- and post-training performances and differences in pre- vs. post-training performance on the one hand and post-onset time on the other. This indicates that spontaneous recovery does not explain all the observed improvements after training. Also, mean scores of both groups as a whole on the Bells test showed a reduction of approximately five omissions after training. This coincides with the maximum test–retest variability in the Machner et al. (2012) study (see also the Section Introduction). This result suggests that patients’ progress can not entirely be ascribed to test–retest variability, although some learning effect may have been present. Our results seem in concordance with previous studies evaluating TSVS (Pizzamiglio et al., 1992; Antonucci et al., 1995; Paolucci et al., 1996). Nevertheless, the inclusion of a no-treatment control group is still recommended for future research. Including a control group might also be useful to rule out the possible role of other rehabilitation treatments that patients receive during the experimental or control training.

As in standardized TSVS, the mere amount of training time might be crucial also in dual task training (Antonucci et al., 1995; Kerkhoff, 1998). Therefore, in future research, increasing the amount of dual task training in the experimental group should be considered. The current training schedule was partly based on the standardized TSVS protocol (Pizzamiglio et al., 1990) and partly on clinical experience. Although it was presumed that patients first should learn the “centering” technique before moving on to the CVRT-TR task in the experimental condition, other training schedules allowing for more dual task practice might be considered. For example, after first introducing the centering technique during two or three training sessions in the fourth week of training using the TSVS digit detection task, the automatization of this skill might be further practiced using the CVRT-TR on a daily basis. Moreover, a repeated evaluation of the patients’ performances with our assessment measures could have been useful. This might have unraveled the presence of a tendency to improve between the first half (equal for both groups) and the second half (different for the two groups) of the training. Moreover, repeated evaluation during training might reveal the time needed for substantial improvement and be useful to chart patients’ progress during different training stages. Although 6 weeks of training may be considered time-consuming, the original TSVS training protocol by Pizzamiglio et al. (1990) envisages 8 weeks of training. Additional driving simulator training beyond 6 weeks might turn out to be necessary to allow the generalization of training results. This would also have minimized the demands on the patients’ impaired abilities in maintaining corrective “top-down” control over spatial attention (Robertson and Manly, 2004).

In order to further evaluate the possible additional effects of dual task training and the design of future VR dual tasks for the training of neglect, it is important to address the issue of the large variability in neglect symptoms and training effects between patients. It may well be worth to evaluate larger groups of patients and to reconsider inclusion criteria for dual task training. For instance, despite the suggestion that it should be possible to also train patients with mild neglect using the CVRT and CVRT-TR, it must be noted that the groups participating in the present study consisted of patients with chronic and moderate to severe neglect. Although the CVRT-TR was, among other things, designed to allow patients with mild neglect to train visual scanning strategies up to a higher level of automation and under more challenging conditions, the inclusion criteria of the present study mostly led to the exclusion of patients with these milder degrees of neglect. Also, the CVRT-TR turned out to be too difficult for some of the participating patients. Two of them even complained that the dual task was unpleasant and had the impression that they were not improving. It might be worthwhile to evaluate which patients might really benefit from dual task training. To this end, data on the location and size of patients’ lesions might be informative and aid in the tailoring of interventions.

Finally, no specific strategies were presently proposed to patients to systematically improve single and dual lane tracking. In future dual task training developments, the design of progressively increasing difficulty levels might be considered, coupled to the formulation of helpful cues and strategies to be learned accordingly. For instance, the addition (and gradual reduction) of spatial cues regarding their actual lateral position and a built-in control or “brake” function might be helpful for patients who have difficulties performing the dual task. For some patients, monitoring (the risk of) errors or omissions and exerting control over the situation by pausing and taking time to scan the environment might be an important strategy to compensate for neglect. Similarly, suitable strategies might be developed for patients suffering from neglect in combination with visual field deficits, who were excluded from the present study.

In conclusion, previous research has pointed out that computerized (dual) tasks may be very useful in the assessment of neglect (Schendel and Robertson, 2002; Bonato and Deouell, 2013). Before any recommendation can be made about the use of these tasks for training, further research is needed. Alongside the abovementioned methodological suggestions, future research might focus on the relationship between ameliorations on dual task performance and the performance on other outcome measures. For example, a robust relationships between CVRT-D performance and measures of mobility, balance, and daily functioning has recently been found (Van Kessel et al., 2012). It would be worthwhile to investigate whether possible training effects on driving simulator tasks might also be reflected in the reduction of neglect symptoms in real-life tasks like walking or (wheelchair) driving.

## Conflict of Interest Statement

The authors declare that the research was conducted in the absence of any commercial or financial relationships that could be construed as a potential conflict of interest.
